# Identification of CHRNB4 as a Diagnostic/Prognostic Indicator and Therapeutic Target in Human Esophageal Squamous Cell Carcinoma

**DOI:** 10.3389/fonc.2020.571167

**Published:** 2020-11-16

**Authors:** Nan Li, Kaisheng Liu, Shaowei Dong, Ling Ou, Jieling Li, Minshan Lai, Yue Wang, Yucheng Bao, Huijie Shi, Xiao Wang, Shaoxiang Wang

**Affiliations:** ^1^ School of Pharmaceutical Sciences, Shenzhen University Health Science Center, Shenzhen, China; ^2^ Shenzhen People’s Hospital (The Second Clinical Medical College, Jinan University; The First Affiliated Hospital, Southern University of Science and Technology), Shenzhen, Guangdong, China

**Keywords:** esophageal squamous cell carcinoma, cholinergic nicotinic receptor subunit, diagnosis, prognosis, CHRNB4, mTOR

## Abstract

Esophageal squamous cell carcinoma (ESCC) is one of the most aggressive malignant tumors and there is a lack of biomarkers for ESCC diagnosis and prognosis. Family subunits of cholinergic nicotinic receptor genes (CHRNs) are involved in smoking behavior and tumor cell proliferation. Previous researches have shown similar molecular features and pathogenic mechanisms among ESCC, head and neck squamous cell carcinoma (HNSC), and lung squamous cell carcinoma (LUSC). Using edgeR, three mutual differentially expressed genes of CHRNs were found to be significantly upregulated at the mRNA level in ESCC, LUSC, and HNSC compared to matched normal tissues. Kaplan–Meier survival analysis showed that high expression of CHRNB4 was associated with unfavorable prognosis in ESCC and HNSC. The specific expression analysis revealed that CHRNB4 is highly expressed selectively in squamous cell carcinomas compared to adenocarcinoma. Cox proportional hazards regression analysis was performed to find that just the single gene CHRNB4 has enough independent prognostic ability, with the area under curve surpassing the tumor-node-metastasis (TNM) staging-based model, the most commonly used model in clinical application in ESCC. In addition, an effective prognostic nomogram was established combining the TNM stage, gender of patients, and expression of CHRNB4 for ESCC patients, revealing an excellent prognostic ability when compared to the model of CHRNB4 alone or TNM. Gene Set Enrichment Analysis results suggested that the expression of CHRNB4 was associated with cancer-related pathways, such as the mTOR pathway. Cell Counting Kit-8, cloning formation assay, and western blot proved that CHRNB4 knockdown can inhibit the proliferation of ESCC cells *via* the Akt/mTOR and ERK1/2/mTOR pathways, which might facilitate the prolonged survival of patients. Furthermore, we conducted structure-based molecular docking, and potential modulators against CHRNB4 were screened from FDA approved drugs. These findings suggested that CHRNB4 specifically expressed in SCCs, and may serve as a promising biomarker for diagnosis and prognosis prediction, and it can even become a therapeutic target of ESCC patients.

## Introduction

Squamous cell carcinomas (SCCs), including esophageal squamous cell carcinoma (ESCC), head and neck squamous cell carcinoma (HNSC), and lung squamous cell carcinoma (LUSC), are among the most common human cancers and are harder to cure than adenocarcinomas (1). Worldwide, esophageal cancer (EC) is the 7th most common type of cancer and the 6th most common cause of cancer-related deaths (2). ESCC is the major histological type of EC in East Asian countries and is one of the most aggressive malignant tumors (3, 4). Surgery alone or in combination with neoadjuvant chemoradiotherapy, adjuvant radiotherapy, and/or adjuvant chemotherapy remains the main curative modality for ESCC (5). However, by the time ESCC is diagnosed, tumors often have already spread throughout the body, meaning patients cannot receive timely treatment. Unfortunately, few sensitive and specific ESCC biomarkers have been clinically validated and can be used routinely. Thus, there is an urgent need to identify novel biomarkers that can be used in the diagnosis and prognosis of ESCC.

The Cancer Genome Atlas (TCGA), which is an effective tool to discover novel cancer targets through high-throughput sequencing techniques, is recognized as the most commonly used and authoritative database. Although ESCC is a common cancer type, the sample numbers in the TCGA database are relatively small (only 92 normal and tumor samples for ESCC). Fortunately, previous genomic analyses suggest that ESCC, HNSC, and LUSC share some common pathogenic mechanisms, such as the MAPK pathway, cell cycle, and JAK-STAT pathway, and therefore, analogous predictive and therapeutic approaches could be considered (6). Additionally, ESCC had a stronger resemblance to HNSC than to esophageal adenocarcinoma (EAC) with respect to some molecular features, such as mRNA expression, DNA methylation, and somatic copy-number alterations data (7). Therefore, we can explore novel biological markers and tumorigenic pathways that would greatly improve therapeutic strategies for ESCC based on the similarity between ESCC, HNSC, and LUSC.

In our recent bioinformatics studies, we found that tobacco and alcohol were correlated with the overall survival of ESCC patients (8). The role of the cholinergic nicotinic receptor genes (CHRNs) family subunits in tobacco use was confirmed in a previous study (9). CHRNs consist of 16 genes (**Table 1**), and their genetic variants may contribute to lung cancer susceptibility (10–12). Research indicated that CHRNs are important therapeutic targets involved in tobacco-associated lung carcinogenesis (13). Besides, cholinergic nicotinic receptors (nAChRs) encoded by CHRNs are involved in the regulation of metabolic processes and cell-cell interactions related to carcinogenesis and tumor-associated inflammation (14). Although nAChRs, which are major factors that contribute to the development of ESCC, are important relevant proteins in the process of smoking, their diagnosis and prognosis roles are still poorly understood (15). Therefore, it is worthwhile to investigate whether CHRNs can be new markers of ESCC.

**Table 1:** The family genes of CHRN.

Symbol	Gene name	Chromosome location	Aliases
CHRNA1	CHRN alpha 1 subunit	2q31.1	ACHRA; ACHRD
CHRNA2	CHRN alpha 2 subunit	8p21.2	
CHRNA3	CHRN alpha 3 subunit	15q25.1	LNCR2; PAOD2
CHRNA4	CHRN alpha 4 subunit	20q13.33	EBN; BFNC
CHRNA5	CHRN alpha 5 subunit	15q25.1	LNCR2
CHRNA6	CHRN alpha 6 subunit	8p11.21	CHNRA6
CHRNA7	CHRN alpha 7 subunit	15q13.3	NACHRA7; CHRNA7-2
CHRNA9	CHRN alpha 9 subunit	4p14	HSA243342; NACHRA9
CHRNA10	CHRN alpha 10 subunit	11p15.4	
CHRNB1	CHRN beta 1 subunit	17p13.1	ACHRB; CHRNB
CHRNB2	CHRN beta 2 subunit	1q21.3	EFNL3; nAChRB2
CHRNB3	CHRN beta 3 subunit	8p11.21	
CHRNB4	CHRN beta 4 subunit	15q25.1	
CHRND	CHRN delta subunit	2q37.1	ACHRD; CMS2A
CHRNE	CHRN epsilon subunit	17p13.2	ACHRE; CMS1D
CHRNG	CHRN gamma subunit	2q37.1	ACHRG

Our colleagues have discovered some useful biomarkers of ESCC by mining the TCGA database (8). Furthermore, this study sought to systematically investigate the expression of CHRNs and its value as a diagnostic and prognostic marker in ESCC, under the auxiliary proof and analysis of data from HNSC and LUSC. Based on bioinformatics analysis and target validation by siRNA-mediated knockdown, our findings suggest that the expression of some CHRNs, especially CHRNB4, is significantly associated with the prognosis of ESCC and might serve as a potential diagnostic/prognostic marker and even as a therapeutic target in ESCC.

## Materials and Methods

### Sample and Data Collection

CHRNs expression data of ESCC, HNSC, and LUSC was obtained from the TCGA database (https://cancergenome.nih.gov/), 92 samples (11 normal samples and 81 tumor samples) from ESCC, 546 samples (44 normal samples and 542 tumor samples) from HNSC, 551 samples (49 normal samples and 502 tumor samples) from LUSC, and 91 samples (11 normal samples and 80 tumor samples) from EAC, respectively. The corresponding clinical information of ESCC patients (95 samples) was also downloaded from the TCGA official website. The mean expression of cervical squamous cell carcinoma (CECC), LUSC, HNSC, stomach adenocarcinoma (STAD), lung adenocarcinoma (LUAD), pancreatic adenocarcinoma (PAAD), colon adenocarcinoma (COAD), and rectum adenocarcinoma (READ) was obtained from OncoLnc (http://www.oncolnc.org).

### Differentially Expressed Genes Analysis

The gene expression data of CHRNs from three datasets, 11 normal cases with 81 tumor cases from ESCC, 44 normal cases with 542 tumor cases from HNSC, and 49 normal cases with 502 tumor cases from LUSC, were included in the DEGs analysis. The edgeR package was used to normalize all the gene expression in TCGA and got the log_2_ fold change (logFC) and *p*-values which were analyzed with the exact test (16). |logFC| > 0.5 and *P <* 0.05 was set as the cut-off value. The heatmap, boxplot, and Venn Diagram packages were used to identify the mutual DEGs of CHRNs in three cancers using R, version 3.5.1 (http://www.r-project.org).

### Survival Analysis of Cancer Patients

According to the expression quantity of each gene in DEGs, we ranked the patients from small to large and divided them into two groups: high expression group and low expression group. To identify the correlation between gene expression and overall survival of patients, we plotted the survival curves using the Kaplan–Meier method and compared two groups using the log-rank test and the “Survival” package by R software. *P <*0.05 was considered statistically significant. The survival time of ESCC HNSC and LUSC patients were compared with GraphPad Prism 5.

### The Expression Analysis

The expression in transcription level from CECC, ESCC, LUSC, HNSC, STAD, LUAD, PAAD, COAD, READ, ESCC, and EAC was analyzed to find out the expression specificity of the core gene.

### Forecast Model Analysis Establishment

We performed the multivariate Cox hazard regression analysis based on the expression of CHRNB4 and the overall survival of patients to establish the forecast model with the help of the survival package. Afterward, we calculated the risk score of every patient according to the formula of our model:

$$\text{Risk score}=\sum_{i=1}^{n}Coef_{i}\times Exp_{i}$$

The receiver operating characteristic (ROC) curve was plotted and the area under curve (AUC) was calculated with the “survivalROC” package to evaluate the capability of distinguishing tumor and normal tissue based on the Risk score. We screened the independent prognostic variables of overall survival (OS) (*p*<0.05) based on univariate and multivariate Cox regression analysis. Nomogram for individual prediction was generated based on the expression of CHRNB4 and independent prognostic risk factors through the R software. Samples were divided into two groups (low- and high-risk groups) on the basis of the median value of risk score, the Kaplan–Meier method was carried out to plot survival curves, and then the two groups were compared using the log-rank test. The performance of the nomogram was validated by assessing the AUC.

### Gene Set Enrichment Analysis

The enriched pathways of CHRNB4 in gene expression levels between high-risk and low-risk groups were analyzed using GSEA (https://www.broadinstitute.org/gsea/index.jsp). Oncogenic signatures gene sets (c6), curated gene sets (c2), and hallmark gene sets (h) were used as references.

### Co-Expression Network Construction

Weighted Gene Co-Expression Network Analysis (WGCNA) was conducted to assess the relationships between the CHRNB4 and other genes in TCGA (|Cor|>0.5). The visual networks were plotted through Cytoscape (http://www.cytoscape.org/, v3.6.1) (17).

### Cell Lines and Cell Culture

KYSE30 and KYSE450 cell lines were bought from Shanghai Institutes for Biological Sciences, Chinese Academy of Sciences (Shanghai, China). Cells were cultured in RPMI 1640 medium (Gibco Life Sciences, USA) supplemented with 10% fetal bovine serum (Gibco Life Technologies), 100 U/mL penicillin (Gibco; Thermo Fisher Scientific, Inc.), and 100 µg/mL streptomycin (Gibco; Thermo Fisher Scientific, Inc.) and were then incubated at 37°C humid incubator containing 5% CO_2_.

### Quantitative Real-Time Polymerase Chain Reaction Analysis

The RNeasy^®^Mini Kit (50) was used to extract total RNA from ESCC cells. GAPDH was the endogenous normalizer. Data was calculated *via* a 2^−ΔΔCt^ method and analyzed on LightCycler^®^96 Instrument, Roche.

### Cell Counting Kit-8 Analysis

Cell viability was analyzed by the CCK-8 assay (Beyotime Institute of Biotechnology, China) as instructed by its manufacturer. Cells were seeded in 96-well plate with 5,000 cells/well, 2,000 cells/well, 1,000 cells/well, and 500 cells/well before incubation for respectively 1, 3, 5, and 7 d. si- negative control (NC) (1pmol) and siRNAs were transfected by Lipofectamine™ RNAiMAX Transfection Reagent according to the protocol (ThermoFisher Scientific). Afterward, 10 µl of CCK-8 solution and equal serum-free medium, replacing the former culture medium, was supplemented to culture the cells at 37°C for 2 h. The absorbance at 450 nm was detected by the microplate reader (Bio-Rad Laboratories Inc., Hercules, CA). Cell viability was calculated by the formula: cell viability ratio (%) = (O.D. NC – O.D. treated)/O.D. NC × 100%.

### Colony Formation Assay

Five hundred cells were seeded in a 6-well plate. After 24 h incubation at 37°C, 25 pmol si-NC and siRNAs were transfected by Lipofectamine™ RNAiMAX Transfection Reagent according to the protocol (ThermoFisher Scientific). Following the 7 d incubation, the medium was removed, and the plates were washed with phosphate-buffered saline. Cells were then fixed with methanol for 15 min and stained with 0.1% crystal violet for 5 min at room temperature. Cell colonies were captured using a scanner.

### Western Blot

RIPA lysis buffer containing protease inhibitor cocktail and phosphatase inhibitor cocktail (Beyotime, Shanghai, China) was used to extract whole proteins from the ESCC cells. The concentration of the protein was determined using a bicinchoninic acid assay kit (Beyotime, Shanghai, China). Afterward, proteins were separated by SDS-PAGE with corresponding gel and transferred onto polyvinylidene fluoride (PVDF) membranes (Millipore, USA). Subsequently, the membranes were blocked in 5% skim milk for 1 h washed with TBST three times every 5 min, and then were incubated with primary antibodies overnight at 4°C. Additionally, the primary antibodies GAPDH (1:3,000 dilution, cat no. 5174S) and Akt (1:1,000 dilution, cat no. 13038) as well as the P-Akt (1:1,000 dilution, cat no. 4691), mTOR (1:1,000 dilution, cat no. 2983), P-mTOR (1:1,000 dilution, cat no. 5536), ERK1/2 (1:1,000 dilution, cat no.2721), and P-ERK1/2 (1:1,000 dilution, cat no. 4903) antibodies were purchased from Cell Signaling Technology. After washing three times with TBST, membranes were incubated with secondary HRP-conjugated antibodies for 1 h at room temperature. Later, blotting bands were detected using Chemiluminescent HRP Substrate (Millipore Corporation, USA) and were visualized with UVP GelStudio PLUS Touch (Analytik Jena, Germany). In the accompanying bar graphs, protein levels were normalized to GAPDH, which was used as a loading control. Results are shown as means ± S.D. of three independent experiments.

### Molecular Docking Analysis

The CHRNB4 protein structure was retrieved from Protein Data Bank (www.rcsb.org) using entry 6PV7. The names and structures of FDA approved drugs were retrieved from the Drug Bank (18). The docking was performed using Hex 8.0.0 (19) with default docking parameters. The docking scores can be interpreted as an interaction energy, and the lower scores represent lower energy, and higher rankings.

### Statistical Analysis

Results were analyzed by R version 3.51 (https://www.r-project.org/) and GraphPad Prism version 5 (GraphPad Software, San Diego, CA, USA). Two-tailed *P*-values of less than 0.05 were defined as significant differences. Experimental data were presented as mean ± SD of at least three independent experiments and the results were analyzed using an unpaired Student’s t-test and Two-way ANOVA. The Pearson correlation test was used in Scatter diagram based on the R package. |Cor|>0.5 was considered statistically significant.

## Results

### Expression of CHRNs in SCCs Patients

**Figure 1:**
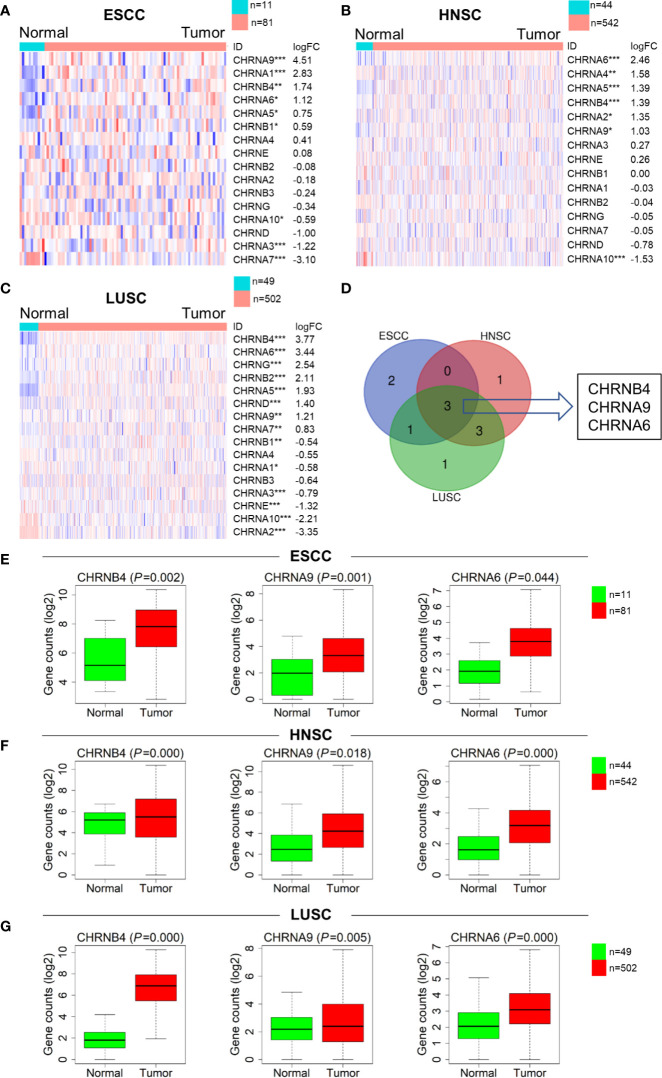
Expression difference of the CHRNs between tumor (ESCC, LUSC, and HNSC) and normal samples. **(A–C)** Heatmaps of the complete CHRN family in 3 cancers in descending order of logFC. The red and blue colors represent high and low expression, respectively. ***P < 0.001; **P < 0.01; *P < 0.05. **(D)** The Venn diagram of the DEGs in ESCC, LUSC, and HNSC**. (E–G)** The box diagrams showed expression levels of 3 genes between normal and tumor in TCGA.

To investigate the expression of CHRNs in ESCC (n = 92), LUSC (n = 546), and HNSC (n = 551), we analyzed the data of the samples mentioned above from TCGA datasets. The expression profiles of genes covering complete CHRNs in the three datasets were further shown by heatmaps (**Figures 1A–C**). We found that there were 6, 7, and 10 genes expressed aberrantly in ESCC, HNSC, and LUSC mRNA expression, respectively. Besides, the Venn diagram demonstrated that there were three mutual DEGs in all three cancers: CHRNB4, CHRNA9, and CHRNA6 (**Figure 1D**). Compared with the normal group, these 3 genes were all markedly upregulated in all three SCCs, with *P*<0.05 (**Figures 1E–G**).

### Survival Analysis and Expression Specificity of CHRNs in SCCs Patients

**Figure 2:**
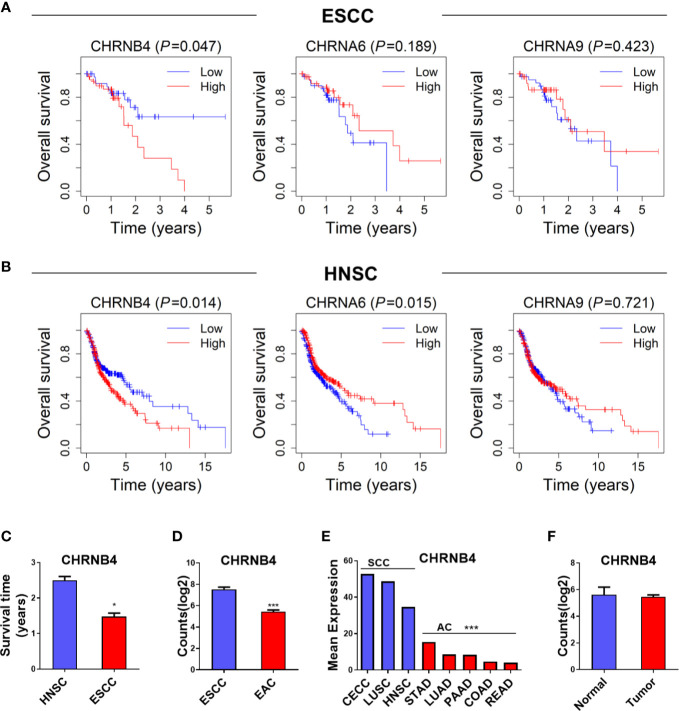
**(A, B)** Survival analysis of 3 CHRN family genes in ESCC, HNSC. **(C)** The survival time between HNSC and ESCC patients. **(D, E)** The expression situation of CHRNB4 in several SCCs and ACs. **(F)** The expression of CHRNB4 between normal and tumor in EAC. ***P < 0.001; *P < 0.05.

To determine the prognostic relevance of the 3 selected CHRN genes in ESCC, LUSC, and HNSC, OS was analyzed using the Kaplan–Meier curves and a log-rank test between the high and low expression groups of each gene, based on TCGA datasets. As shown in the survival curves, the high expression of CHRNB4 was significantly correlated with the low survival rate in ESCC and HNSC patients, while CHRNA6 was only statistically significant in HNSC patients. CHRNA9 was not significantly different in any of the three databases (**Figures 2A, B**, **Supplementary Figure 1**). Moreover, the survival time of HNSC patients was longer than that of ESCC patients, which means ESCC is more malignant than HNSC and therefore prompts a more urgent need for diagnosis markers (**Figure 2C**).

Because CHRNB4 seems to be a very critical gene according to the expression and survival analysis, we then explored its expression specificity in different cancer types. We found the expression of CHRNB4 was higher in cancer with SCCs including CECC, LUSC, HNSC, and ESCC than in adenocarcinomas (AC) such as STAD, LUAD, PAAD, COAD, READ, and EAC (**Figures 2D, E**), suggesting that CHRNB4 was significantly and specifically upregulated in SCCs compared to ACs. Further, compared with the upregulation of CHRNB4 in ESCC, it was not significantly different in EAC (**Figure 2F**, *P >* 0.05). The results indicate that CHRNB4, which was significantly correlated with patient survival in SCCs, is also specifically highly expressed in SCCs but not in ACs.

### CHRNB4-Based Forecast Model and Prognostic Nomogram to Predict the Prognosis of ESCC

**Figure 3:**
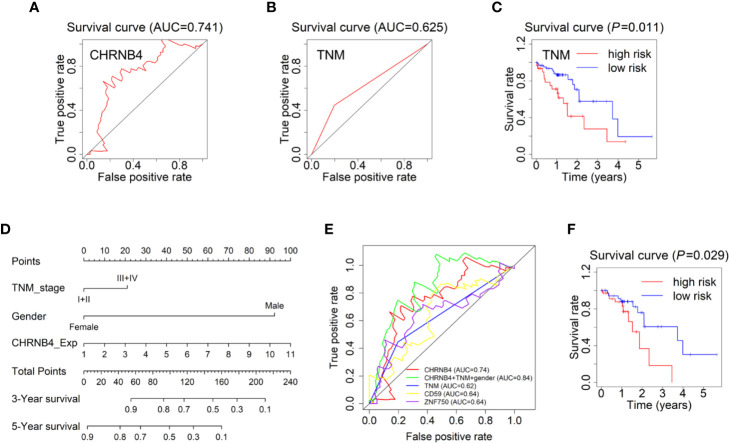
Forecast models and survival nomogram to predict the survival of ESCC patients. **(A)** The 3 year-ROC Forecast models of CHRNB4 in ESCC. **(B, C)** ROC and survival curves of the models according to TNM staging in ESCC-TCGA. **(D)** ESCC survival nomogram combined the TNM stage, gender, and the expression of CHRNB4. **(E)** The prognosis ability of our CHRNB4, prognostic nomogram, TNM, and two published biomarkers (CD59 and ZNF750). **(F)** Survival curves of the nomogram.

The survival ROC curve is used to show the predictive accuracy of the gene signature, and the AUC value represents predictive power. In the ROC curve, the red line represents the sensitive curve, while black represents the identified line. The AUC of CHRNB4 reached 0.740 (**Figure 3A**), which was obviously higher than that of the ESCC-TNM Stage, with the survival curve showing significantly differences between high- and low-risk groups (AUC=0.625, *P* = 0.011, **Figures 3B, C**). It is worth mentioning that the TNM forecast model is the classic model in clinical use nowadays (20, 21). According to the results mentioned above, the single CHRNB4 can become a novel, reliable, and independent prognostic marker for better management of ESCC, with sufficient predictive ability, stronger than the traditional TNM-based model.

Univariate analysis demonstrated that the TNM stage and gender were independent risk factors for OS in TCGA, which is consistent with our previous results (8). The expression of CHRNB4 is an independent risk factor, while the N stage was included in TNM stage. Therefore, to develop a more accurate prediction model, we further established a nomogram integrating the CHRNB4 expression, TNM stage, and gender (**Figure 3C**). The C-index for OS prediction was 0. 633 (95% CI, 0.557 to 0.709). **Figure 3D** and **3E** showed the prognosis ability of our CHRNB4 and prognostic nomogram (AUC=0.84) are as good as or even better than TNM, CD59, and ZNF750 (AUC=0.64, 0.64, respectively), which were previously published biomarkers in ESCC (22, 23). The survival curve for the probability of survival of the nomogram is shown in **Figure 3F** (*P* =0.03). Collectively, our forecast models based on CHRNB4, TNM stage, and gender are promising in ESCC patients’ prognosis.

### GSEA and Co-Expression Network Analysis of CHRNB4

**Figure 4:**
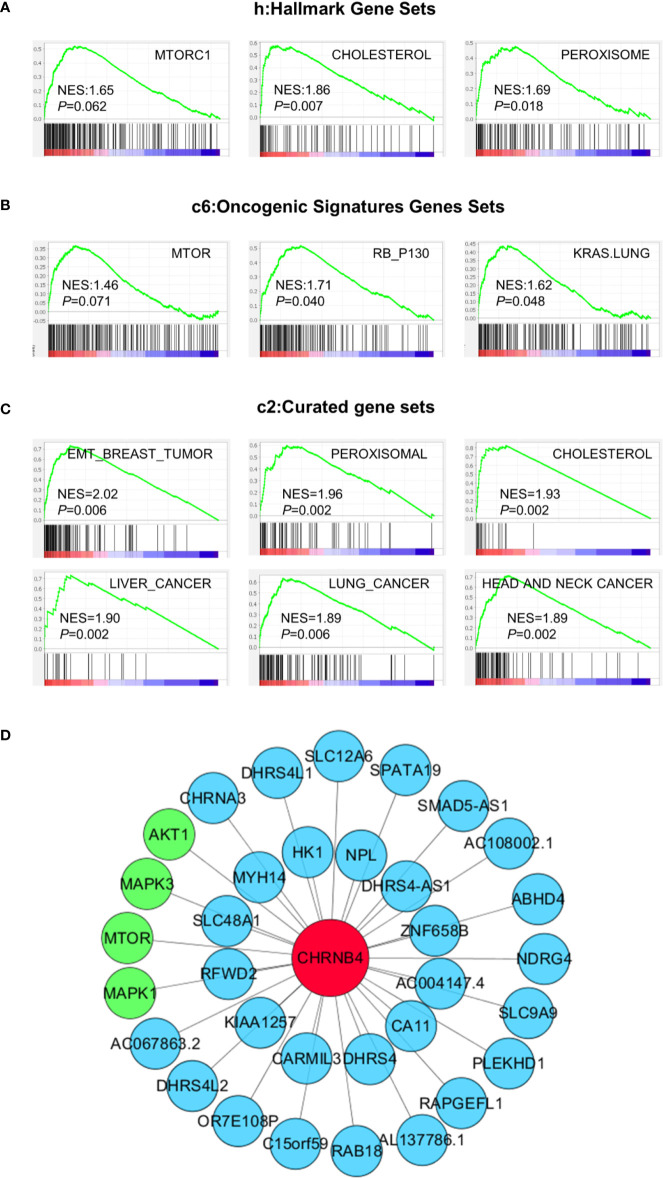
GSEA results based on the expression of CHRNB4 in TCGA and Co-expression pictures of CHRNB4. **(A–C)** Significantly enriched gene sets were obtained according to hallmark gene sets (h), oncogenic signatures gene sets (c6), and curated gene sets (c2). The gene sets highly correlated with tumor were presented. **(D)** The pictures were shown that the relativity between the gene CHRNB4 and the relevant genes. The red nodes are CHRNB4. The green nodes are the genes of the mTOR signaling pathways and blue nodes are many other less well-known genes.

To investigate the biological pathway and progression involved in ESCC pathogenesis of CHRNB4, GSEA analysis was performed on the ESCC tumor samples in the TCGA database. The cancer-associated gene sets including hallmark gene sets (h), oncogenic signatures gene sets (c6), and curated gene sets (c2) in the GSEA website were used as references. According to h gene sets (**Figure 4A**), mTORC1 (NES = 1.65, *P* = 0.062), cholesterol (NES = 1.86, *P* = 0.007), and peroxisome (NES = 1.69, *P* = 0.018) were enriched in CHRNB4-high group. In the top 20 pathways of CHRNB4 in c6 (**Figure 4B**), gene sets of RB (NES = 1.71, *P* = 0.040), KRAS (NES = 1.62, *P* = 0.048), and mTOR (NES = 1.46, *P* = 0.071) showed correlation with the high-risk group. In c2 gene sets (**Figure 4C**), EMT (NES = 2.02, *P* = 0.006), peroxisomal (NES = 1.96, *P* = 0.002), cholesterol (NES = 1.93, *P* = 0.002), liver cancer (NES = 1.90, *P* = 0.002), lung cancer (NES = 1.89, *P* = 0.006), and head and neck cancer (NES = 1.89, *P* = 0.002) were significantly upregulated. These results indicate that the expression of CHRNB4 is closely related to many cancer-related hallmarks and signaling pathways such as mTOR.

The co-expression network might help to understand the interconnections of CHRNB4 with other hub genes. WGCNA was conducted to identify genes that were co-expressed with CHRNB4. The co-expression networks of the CHRNB4 in TCGA and other genes were visibly drawn using the Cytoscape software (**Figure 4D**). CHRNB4 were displayed as bigger red nodes. The other nodes represented the co-expressed genes. We mapped the network by sorting the correlation coefficients and selecting co-expressed genes, which are most relevant to the CHRNB4 (|Cor| > 0.5). As shown in the network map, CHRNB4 are closely related to mTOR signaling pathways (green nodes) including mTOR, AKT1, MAPK1, MAPK3, and many other less well-known genes (blue nodes), suggesting that the family of genes has not been well researched.

### Influence of CHRNB4 Knockdown on ESCC Cell Growth

**Figure 5:**
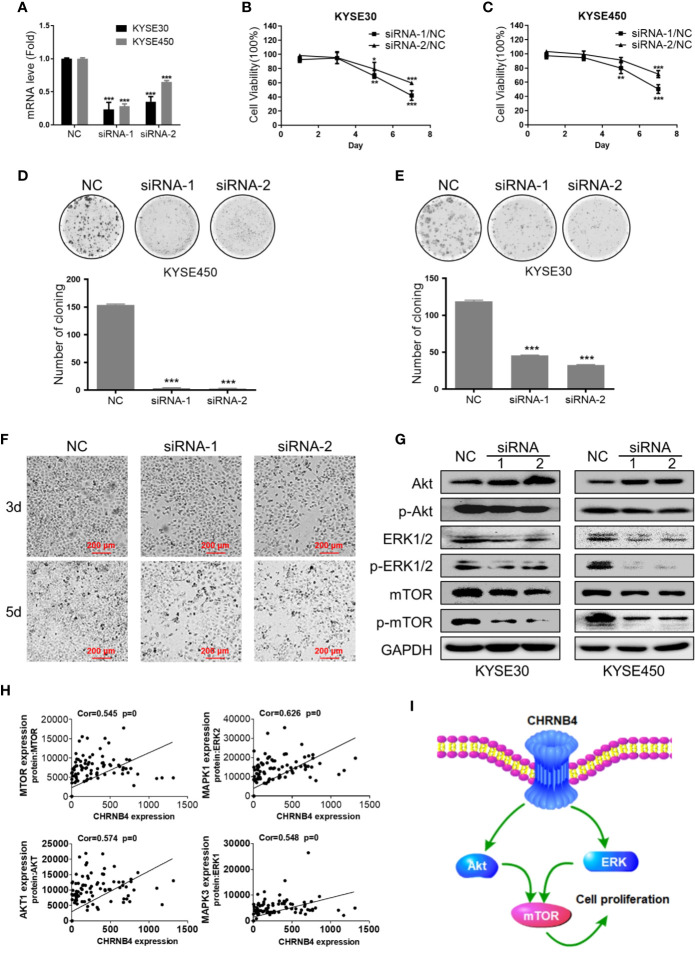
ESCC cell growth after CHRNB4 knockdown by siRNA. **(A)** The interference efficiency of KYSE30 and KYSE450 treated with siRNA**. (B, C)** The cell viability of KYSE30 and KYSE450 treated with siRNA was detected at 1, 3, 5, and 7 d at an optical density of 450 nm. **(D, E)** Pictures of colony formation assay of the 3 groups in KYSE450 and KYSE30 cells, respectively. Histograms of colony numbers of each group in KYSE450 and KYSE30 cells, respectively. **(F)** The cell morphology under the treatment with siRNA at 3 d, 5 d of KYSE30. **(G)** Protein levels of Akt, P-Akt, mTOR, P-mTOR, ERK 1/2, and P- ERK 1/2 were detected by western blot. **(H)** The pictures displayed the correction between CHRNB4 and genes associated with protein regulated mTOR. **(I).** CHRNB4 potential regulation mechanism speculates in the tumorigenesis progression of ESCC. Data are the means ± SD of three replicates. ***P < 0.001; **P < 0.01; *P < 0.05.

From the above data of differential expression and prognosis, CHRNB4 might be the most valuable gene among all the CHRN genes in ESCC. Therefore, in the target validation experiment, we tried to verify whether knockdown of CHRNB4 by siRNA affects ESCC cell proliferation and the mTOR singling pathway enriched by the above GSEA analysis. We first successfully proved the interference efficiency of two siRNA by testing its mRNA level with qRT-PCR in KYSE30 and KYSE450 (**Figure 5A**). As expected, knockdown of CHRNB4 significantly inhibited the cell viability of KYSE30 and KYSE450 at 5 and 7 d (P < 0.05, **Figures 5B, C**). The colony formation assays also revealed that compared to the NC, the colony numbers in the siRNA-treated group were significantly decreased in KYSE450 and KYSE30 cells, respectively (**Figures 5D, E**). Cell morphology indicated that cell numbers of KYSE30 under the treatment with siRNA at 3 d, 5 d were significantly decreased respectively (**Figure 5F**). Moreover, knockdown of CHRNB4 by siRNA seemingly enhanced expression of Akt and reduced expression of Phospho-Akt, mTOR, Phospho-mTOR, ERK1/2, and Phospho- ERK 1/2 (**Figure 5G**, **Supplementary Figure 2**), suggesting that the pathways of mTOR were inhibited. Our bioinformatic results by WGCNA analysis also proved that the core genes of the mTOR pathway, including AKT1, MAPK1, MAPK3, mTOR, encoded proteins Akt, ERK 2, ERK 1, and mTOR, which are closely positively correlated with the expression of CHRNB4 (**Figure 5H**). Together, the above results revealed that CHRNB4 influences the proliferation of ESCC cells through the mTOR signaling pathways enriched by GSEA (**Figure 5I**).

### Molecular Docking Screening Potential Drugs Against CHRNB4

**Figure 6:**
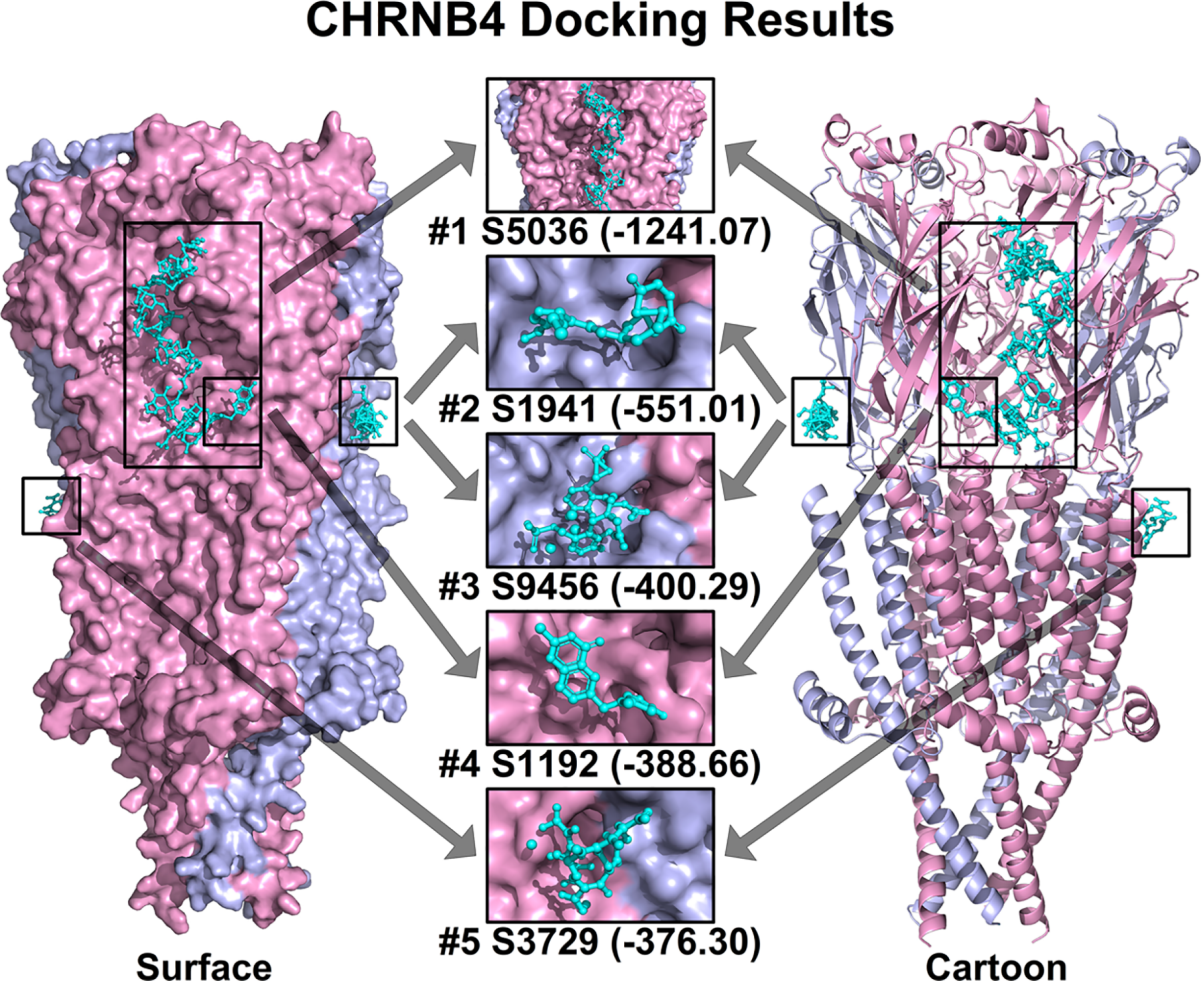
Top 5 ranked small molecules in CHRNB4 docking analysis. The protein structure of CHRNB4 is illustrated in surface format (left) and cartoon format (right) with different chains colored in pink and light blue. The top 5 ranked small molecules in docking analysis together with their docking scores are colored in cyan and highlighted in rectangular. (S5036, Nisin; S1941, Enalapril maleate; S9456, Mangafodipir Trisodium; S1192, Raltitrexed; S3729, Iron sucrose).

Based on all the aforementioned analysis, we hypothesized that CHRNB4 could be a potential target of cancer therapy. Thus, we conducted structure-based molecular docking, and potential modulators against CHRNB4 were screened from FDA approved drugs. The docking was performed using Hex 8.0.0 with default docking parameters. The top 5 ranked small molecules, including Nisin, Enalapril maleate (Vasotec), Mangafodipir Trisodium, Raltitrexed (Tomudex), and Iron sucrose in CHRNB4 docking analysis, are shown in **Figure 6**. Also, the top 100 ranked small molecules based on docking results were listed in **Supplementary Table 1**.

## Discussion

The diagnosis of cancer is very helpful to improve the quality of life for patients. In many other cancer types, the analysis of BRCA1/2 mutation has been used in clinical practice as a predictive marker for breast cancer and effectively improves the 5-year survival rate of patients (24, 25). Difficult in early diagnosis leads to poor prognosis of ESCC patients, which can be improved by using effective biomarkers. Previous studies found that several factors such as the genomic methylation site, the expression of heterogeneous nuclear ribonucleoprotein H1 (HNRNPH1), and miR-153 are associated with OS but not used for clinical application as markers in ESCC (26–28). Therefore, it is urgently needed to identify novel markers for ESCC. nAChRs, the members of a superfamily of ligand-gated ion channels, can affect the progression of cancer patients through tumor-related pathways. Considering that, it is significant to utilize the links between these cancers to investigate diagnostic and prognostic value using experimental validation and bioinformatics methods with a large cohort systematically in TCGA.

As reported by previous research, there were some common pathogenic mechanisms and molecular features between ESCC, HNSC, and LUSC (6). In our study, we identified three upregulated genes (CHRNB4, CHRNA6, and CHRNA9) of the whole CHRNs between tumor tissues and normal controls in three kinds of SCCs. Consistent results showed that CHRNB4 was upregulated and more frequent in squamous cell carcinoma than in LUAD (29). Consistently, some researchers have studied the diagnostic and prognostic value of partial CHRNs (only 8 genes) in ESCC and found that CHRNB4 expression was higher in tumor samples than in both the matched surrounding mucosa and esophagus samples from healthy individuals (30). Some scholars also concluded that CHRNA9 was similarly overexpressed in HNSC (31), and the expression of CHRNA6 was significantly higher in molecular features of non-small cell lung cancer (32). In terms of the researched literature, we firstly found that CHRNB4 and CHRNA6 were upregulated in ESCC and HNSC tissues, while CHRNA9 was firstly found upregulated in ESCC and LUSC tissues compared to normal tissues. The comparison of the expression of CHRNB4 in SCCs and ACs suggested that CHRNB4 is selectively overexpressed in SCCs, which is an interesting finding we first found. From our studies, it is necessary to diagnose and treat of ESCC and EAC patients separately. Therefore, it is strongly possible that the expression of CHRNs, especially the 3 aberrantly expressed genes, can be used as potential biomarkers for diagnosis in ESCC, LUSC, and HNSC.

We then performed survival analysis with the 3 overexpressed genes in three SCCs and identified the expression of CHRNB4 was significantly correlated with survival in ESCC and HNSC. In cell proliferation experiment, we also found that knockdown of CHRNB4 by siRNA dramatically restrained the proliferation in ESCC cells, suggesting that CHRNB4 may significantly and independently affect the patient’s survival by changing the proliferation rate of cancer cells. Multivariate Cox regression analysis, which we performed with CHRNs so far relying on large TCGA cohorts, demonstrated that CHRNB4 was an independent risk factor predicting unfavorable OS in ESCC with AUC of 0.74. The predictive ability of a single CHRNB4 gene was considered meaningful because it is generally believed that AUC makes sense when it exceeds 0.6 (33). The results mentioned above dementated that CHRNB4 has an independent and specific prognostic value, which is better than the TNM stage (AUC=0.625). Moreover, the prognostic nomogram model combined CHRNB4 and clinical parameters can reach higher (AUC = 0.84). Therefore, the CHRNB4-based models with excellent prognostic accuracy might become a better alternative for clinical prognosis in ESCC.

To investigate the potential mechanism of CHRNB4 in ESCC prognosis, we grouped the samples according to the expression of CHRNB4 as the input in GSEA and substantiated that CHRNB4 might be involved in mTOR, Cholesterol, and other signaling pathways. Akt and ERK 1/2 are upstream regulatory proteins of the mTOR signaling pathway. The Akt/mTOR is a signaling pathway in mammal cells that coordinates important cell activities (34). The components of the Akt/mTOR pathway were overexpressed and activated in ESCC (35). West et al. showed that activation of nAChRs resulted in downstream activation of the Akt pathway (36). ERK1/2 helped to confer cells resistance to cisplatin-induced apoptosis and promoted cancer cell survival in ESCC (37). In addition to the mTOR pathway, Cholesterol is also enriched in hallmark gene sets which were reported as a potent modulator of nAChR (38). Nicotine contributes to tumorigenesis through the stimulation of nAChRs in HNSC, which is consistent with our GSEA results in curated gene sets (39). Consistent research showed CHRNB4 knockdown led to reduced proliferation and propensity to form colonies in lung cancer cells (40). What is more, our *in vitro* experiments showed that knockdown of CHRNB4 inhibited the growth of ESCC cells and the result of western blot validated that the potential mechanism was the Akt/mTOR and ERK1/2/mTOR signaling pathway, which play an essential role in ESCC progression.

In the co-expression gene network, close related genes surrounding CHRNB4 are given. In this map, CHRNB4 are closely related to mTOR signaling pathways including mTOR, AKT1, MAPK1, MAPK3, and many less well-known genes, suggesting that CHRNB4 are not well researched. It was reported that CHRNB4 were relevant to the solute carrier family members, such as SLC9A9, SLC12A6, and SLC48A1, which may be prognostic predictors in ESCC (41). CHRNB4 was also associated with long noncoding RNA, such as DHRS4-AS1, DHRS4L2, DHRS4L1, and SMAD5-AS1, which functions by affecting the proliferation, invasion (epithelial-mesenchymal transition), cell cycle progression and the apoptosis of cancer cells (42, 43). These genes mentioned in the previous references and the visual network can provide guidance on how the CHRNB4 influences the hub genes leading to accelerating the progress of ESCC.

In the network map, we also found that CHRNB4 and CHRNA3 are closely related. Consistent studies showed that CHRNB4, CHRNA3, and CHRNA5 are grouped in a cluster on chromosome 15q24 (44). CHRNB4 is the core gene of the CHRNA5-CHRNA3-CHRNB4 cluster, and the core role of this cluster of nicotinic acetylcholine receptor subunit genes in tobacco use was confirmed in the previous study (9). Besides, it is well-known that the high risk of heavy drinking is often associated with heavy smoking (45). In our recent studies, we also found that the patients with tobacco or alcohol use had a higher risk of getting ESCC (HR = 1.94, and HR = 2.16, respectively) while tobacco and alcohol were modestly correlated with OS of ESCC patients (8). Therefore, we believe that here we provide evidence for the high expression of CHRNB4 caused by tobacco and alcohol, thereby inducing the formation of squamous cell carcinoma through cancer-related pathways such as mTOR.

Structure-based molecular docking is conceivably the most reliable and widely implemented approach in the early phase of drug discovery (46). In our docking results, the top 5 ranked small molecules including Nisin, Enalapril maleate (Vasotec), Mangafodipir Trisodium, Raltitrexed (Tomudex), and Iron sucrose had high docking score with CHRNB4 protein. However, the literature about these drugs and CHRNB4 is extremely scarce, indicating that activity-based high-throughput screening of these potential drugs may be as important as virtual docking and need systematic research in the future.

The contribution of CHRNs to SCCs progression has gradually attracted people’s attention but still with some difficulties. We have listed some novel evidence by means of bioinformatics analysis and cell experiments, but our study still has some limitations. For instance, the small sample size of the ESCC database, and a more comprehensive TCGA database with enough ESCC patient samples should be worth looking forward to in the future. Aside from it, we performed our study at the level of mRNA but not protein because of the immature proteomics technology. Immunohistochemical verification of clinical specimens may be as important as the cell experiments, but it lacks effective commercial antibodies of CHRNB4, making it difficult to detect its protein level. Progress is not too fast because people have not realized the importance of CHRNs in cancer progression before. Once speed limiting conditions are optimized, we believe that the research progress of CHRN family genes in diagnosis and treatment of SCCs will be greatly accelerated.

The results of this study indicate that CHRNs especially CHRNB4 presents the potential value of the diagnosis and prognosis of ESCC. CHRNB4, CHRNA6, and CHRNA9 were identified as DEGs with promising diagnostic value in SCCs. In addition, the CHRNB4-based prediction model as well as nomogram integrating CHRNB4, TNM stage, and gender are especially promising in predicting ESCC patients’ OS. In terms of the underlying mechanism, CHRNB4 might be associated with the mTOR pathway during the progression of ESCC. Thus, the CHRN family might be a potential diagnostic/prognostic marker and a potential therapeutic target for ESCC prevention and treatment.

## Data Availability Statement

The original contributions presented in the study are included in the article/**Supplementary Material**. Further inquiries can be directed to the corresponding author.

## Author Contributions

NL, KL, and SW conceived and designed the study. NL and XW analyzed the data and prepared the manuscript. SD, LO, and JL performed molecular docking studies and cell experiments. ML, YW, YB, and HS participated in the study design and data analysis. All authors contributed to the article and approved the submitted version.

## Funding

This research was funded by the National Natural Science Foundation of China (82073937), Natural Science Foundation of Guangdong Province (2018A030313122), Shenzhen Science and Technology Project (JCYJ20180305163658916, JCYJ20180228175059744), Shenzhen Key Medical Discipline Construction Fund (SZXK059), and Shenzhen Healthcare Research Project (SZBC2018007).

## Conflict of Interest

The authors declare that the research was conducted in the absence of any commercial or financial relationships that could be construed as a potential conflict of interest.
