# Supplementary figures and images for: Identification of CHRNB4 as a Diagnostic/Prognostic Indicator and Therapeutic Target in Human Esophageal Squamous Cell Carcinoma
Source: Front Oncol. 2020 Nov 16;10:571167. doi: 10.3389/fonc.2020.571167 (PMC7701245; doi:10.3389/fonc.2020.571167)

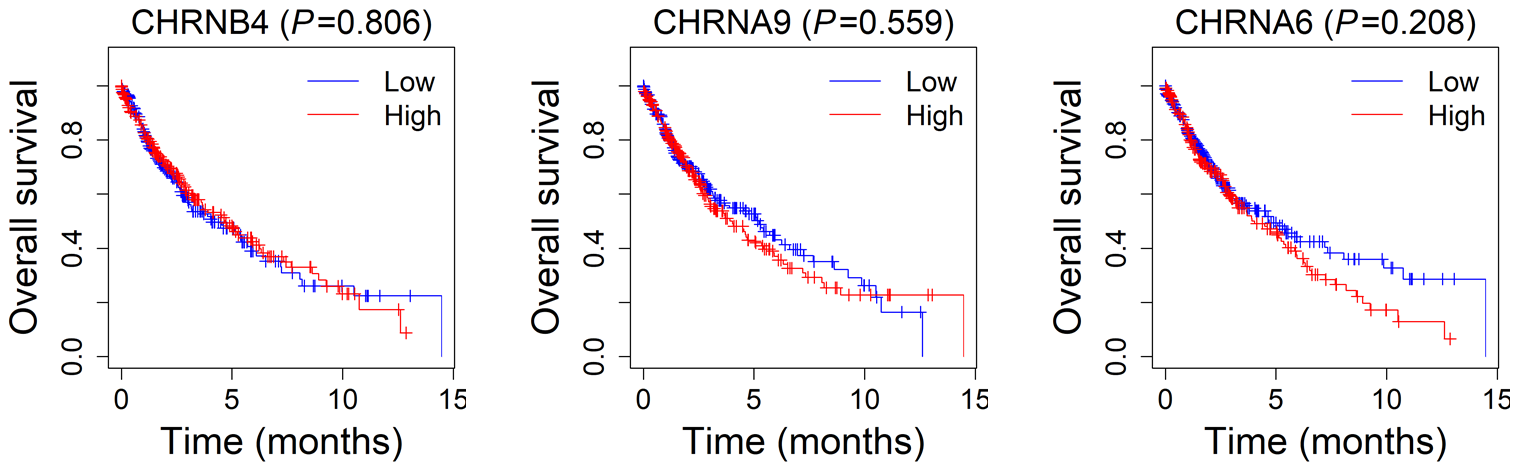

Supplement: Supplementary file 1 [file Image_1.tif]

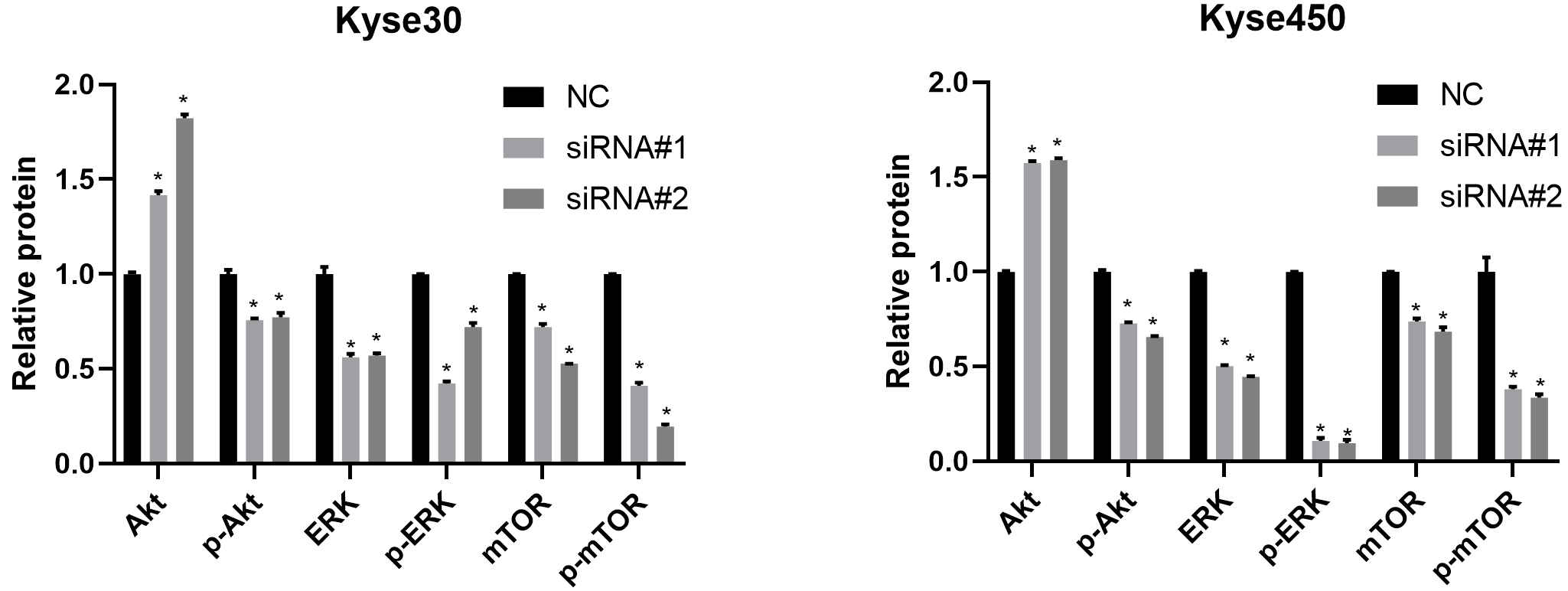

Supplement: Supplementary file 2 [file Image_2.tif]
